# Electric Fields at Breast Cancer and Cancer Cell Collective Galvanotaxis

**DOI:** 10.1038/s41598-020-65566-0

**Published:** 2020-05-26

**Authors:** Kan Zhu, Nicholas R. Hum, Brian Reid, Qin Sun, Gabriela G. Loots, Min Zhao

**Affiliations:** 1 Institute for Regenerative Cures, Departments of Dermatology, Department of Ophthalmology & Vision Science, School of Medicine, University of California, Sacramento, CA 95817 USA; 2 Physical and Life Sciences Directorate, Lawrence Livermore National Laboratory, Livermore, CA 94550 USA; 3 University of California, Merced, School of Natural Sciences, Merced, CA 94550 USA; 4 School of Life Science, Yunnan Normal University, Yunnan, China

**Keywords:** Breast cancer, Cellular motility, Collective cell migration

## Abstract

Cancer growth interferes with local ionic environments, membrane potentials, and transepithelial potentials, resulting in small electrical changes in the tumor microenvironment. Electrical fields (EFs) have significant effects on cancer cell migration (galvanotaxis/electrotaxis), however, their role as a regulator of cancer progression and metastasis is poorly understood. Here, we employed unique probe systems to characterize the electrical properties of cancer cells and their migratory ability under an EF. Subcutaneous tumors were established from a triple-negative murine breast cancer cell line (4T1), electric currents and potentials of tumors were measured using vibrating probe and glass microelectrodes, respectively. Steady outward and inward currents could be detected at different positions on the tumor surface and magnitudes of the electric currents on the tumor surface strongly correlated with tumor weights. Potential measurements also showed the non-homogeneous intratumor electric potentials. Cancer cell migration was then surveyed in the presence of EFs *in vitro*. Parental 4T1 cells and metastatic sublines in isolation showed random migration in EFs of physiological strength, whereas cells in monolayer migrated collectively to the anode. Our data contribute to an improved understanding of breast cancer metastasis, providing new evidence in support of an electrical mechanism that promotes this phenomenon.

## Introduction

Metastasis accounts for ~90% of mortality in breast cancer patients^1,2^. The last few decades have seen significant progress in understanding genetic, molecular and signaling mechanisms underpinning cancer cell migration. Despite this knowledge and implementation of advanced detection technologies, the prevalence of metastatic breast cancer at initial diagnosis has remained stagnant since 1975 in the United States^3–6^. While cancer was long considered a disease defined and driven by genetic evolutions which were mapped to the signaling pathways that regulate cell growth or motility^7–9^, increasing evidence indicates that the maintenance and expansion of malignant cells also strongly depend on external signals from the tumor microenvironment^10–13^.

Due to differences in metabolism and segregation of ions, local electrical properties changed and thus induced small direct current electrical fields naturally in live tissues. It has been shown to closely associate with cancer growth and other biological processes, such as wound healing. For instance, electric currents/fields at wounds are readily measurable and can persist from hours to weeks^14,15^. Similarly, cancer growth interferes with local ionic environments, membrane and transepithelial potentials thus producing local electric fields^16,17^. Outward current can be detected at the surface of tumor, and this electrical current is significantly greater than the one measured at the surface of intact epithelium^18^. Besides, it’s been hypothesized that measurement of electrical potential at the skin surface of new growth has potential to provide a reliable index for breast cancer diagnosis and may help to differentiate between malignant and benign growths^19,20^.

Previous studies by our group and others have demonstrated that galvanotaxis/electrotaxis, directional cell migration in response to extracellular electric gradients, is a powerful mechanism affecting motility and directionality of many cell types^14,21^. It has been demonstrated that cancer cells change their migratory patterns in electric fields of physiological strength^22^. Tumor cells from the brain, prostate and lung have all shown galvanotaxis responses^23–25^, therefore it is likely that most cancer cells exhibit some level of galvanotaxis^14,21,26,27^. Moreover, the galvanotactic responses of cancer cells may correlate with their metastasis capability. The highly invasive lung cancer subline CL1-5 displayed anodal galvanotaxis with increased cell motility, whereas the less invasive subline, CL1-0, displayed low galvanotaxis^28^. Highly metastatic breast cancer cells have been shown to respond to EFs with significantly higher speed and migration directionality than less metastatic cells^26,27^. Electric fields thus may be a fundamental, yet poorly understood, regulator of cancer progression^29–31^.

Herein, to illustrate the tumor endogenous EFs, we established a cell line derived tumor allograft (CDA) model in NSG mouse with the murine mammary carcinoma cell line (4T1) and systematically measured the electric currents and IntraTumoral potential (ITP) at subcutaneous CDA tumors *ex vivo*. The galvanotaxis response of 4T1 cells in EFs of physiological strength was also tested *in vitro*. Lastly, cancer sublines derived from 4T1 metastases to various organs were established and also evaluated for galvanotaxis activity in an EF. Our results demonstrated that electric fields naturally exist at the CDA tumor surface, and 4T1 cells respond to the EFs of physiological strength in monolayer but not in isolation. Metastatic sublines also showed significant galvanotactic movement in EFs with subtle differences.

## Results

### Electrical current measured at tumor’s surface

**Figure 1:**
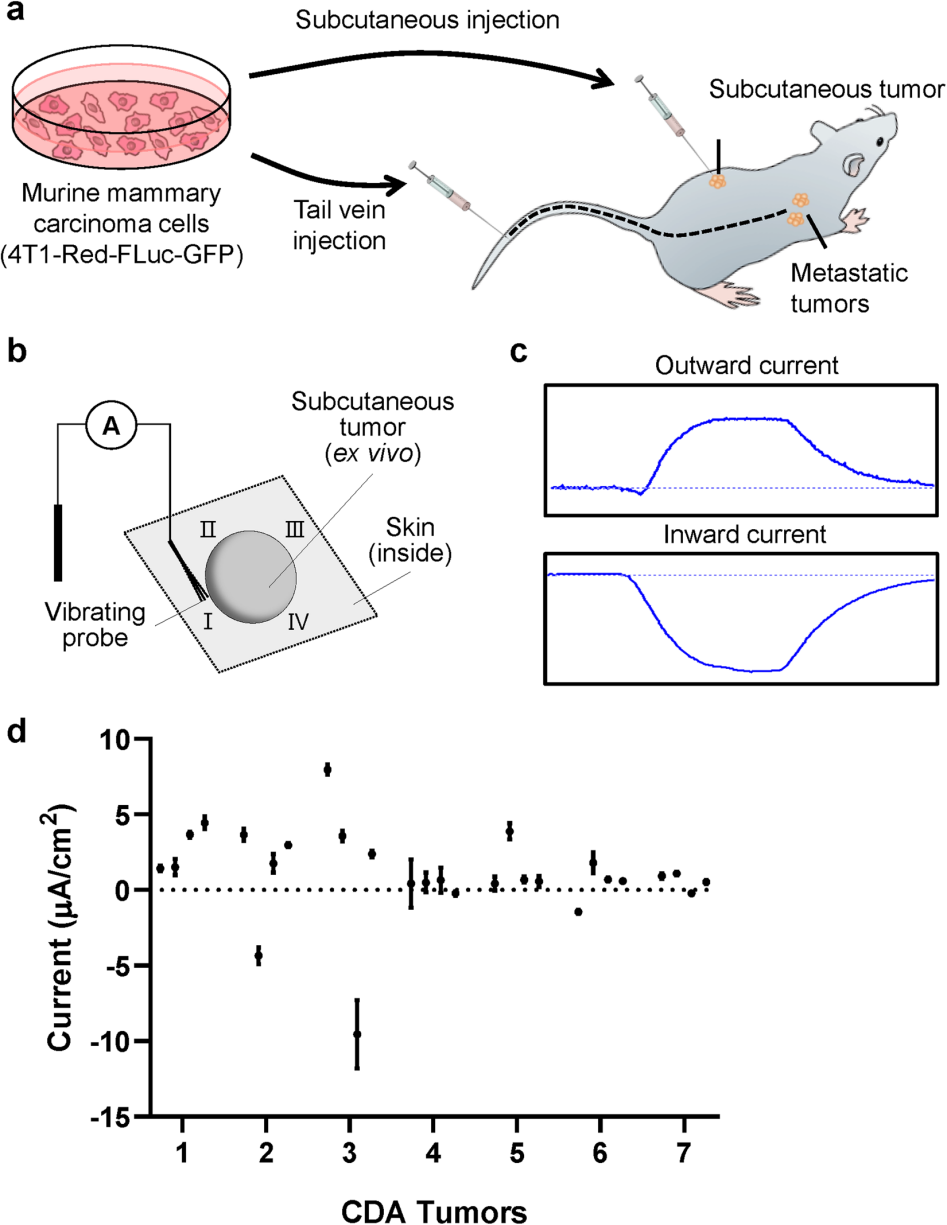
Non-invasive measurement of electrical currents at tumors *ex vivo*. (**a**) Cell Line Derived Tumor Allograft (CDA) Mouse Model (**b**) Schematic drawing of the electrical current measurement using vibrating probe. (**c**) Representative measurements of the outward and inward currents. (**d**) Measurements made at four cardinal points of the tumor surface. Three replicate measurements were made at each point, data are shown as mean ± SEM of each point.

First, the vibrating probe was used to map the electrical currents in transgenic tumors. 1 × 10^5^ 4T1-Red-FLuc-GFP cells were delivered subcutaneously in the dorsal flank of NSG mice and tumors were allowed to establish for 3–4 weeks (Fig. 1a). Dissected tumors were immersed in mouse Ringer’s solution and four cardinal points surrounding the tumor were measured to determine the currents (Fig. 1b). Representative measurements of currents measured are shown in Fig. 1c. Signals greater than the background level indicate the outward currents, while signals lower than the background indicate inward currents. The average current measurement from seven subcutaneous tumors was 2.21 µA/cm^2^ (Fig. 1d). Most tumors had inward and outward currents, suggesting a circuit of current flowing in and out of the tumors and which may be related to tumor growth or polarization. Plotting all tumors together showed a significant linear correlation of current magnitude with tumor weight (r^2^ = 0.83, *P* = 0.004; Supplemental Fig. 1). These results revealed that tumor indeed generate an electric field at the tumor surface, and the current intensity appears to increase as the tumors increase in size.

### 4T1 tumors produce heterogeneous intratumor electric potential

**Figure 2:**
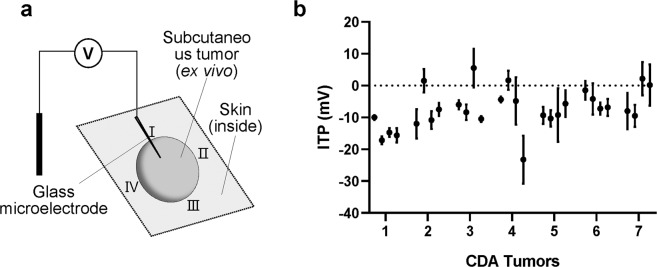
IntraTumoral potential (ITP) measurements using glass microelectrode. (**a**) Schematic drawing of the ITP measurement using glass microelectrode. (**b**) Measurements made at four cardinal points of each tumor. Three replicate measurements were made at each position, data are shown as mean ± SEM of each point.

We next used glass microelectrodes to detect the ITP difference at the same positions of the same tumor. For the ITP measurement, the tumor surface needs to be impaled by the glass electrode tip with a diameter of about 1-2 µm to detect the potential difference between the outside surface and inside of the tumor (Fig. 2a). As shown in Fig. 2b, the ITP measurements showed a similar pattern with the current measurements. Majority of the measurements showed ITP varied from 1.5 mV to 23.25 mV across ~50 µm with the negative inside while 5 isolated positions from 4 tumors generated significant ITP with positive inside. This data supports the idea that the electrical property variations at different parts of the tumor may result in the endogenous EFs that flowing inside and outside of the tumors, which may affect cell migration behavior and ultimately contribute to cancer metastasis.

### Cancer cells showed robust and stronger galvanotaxis collectively than cells in isolation

**Figure 3:**
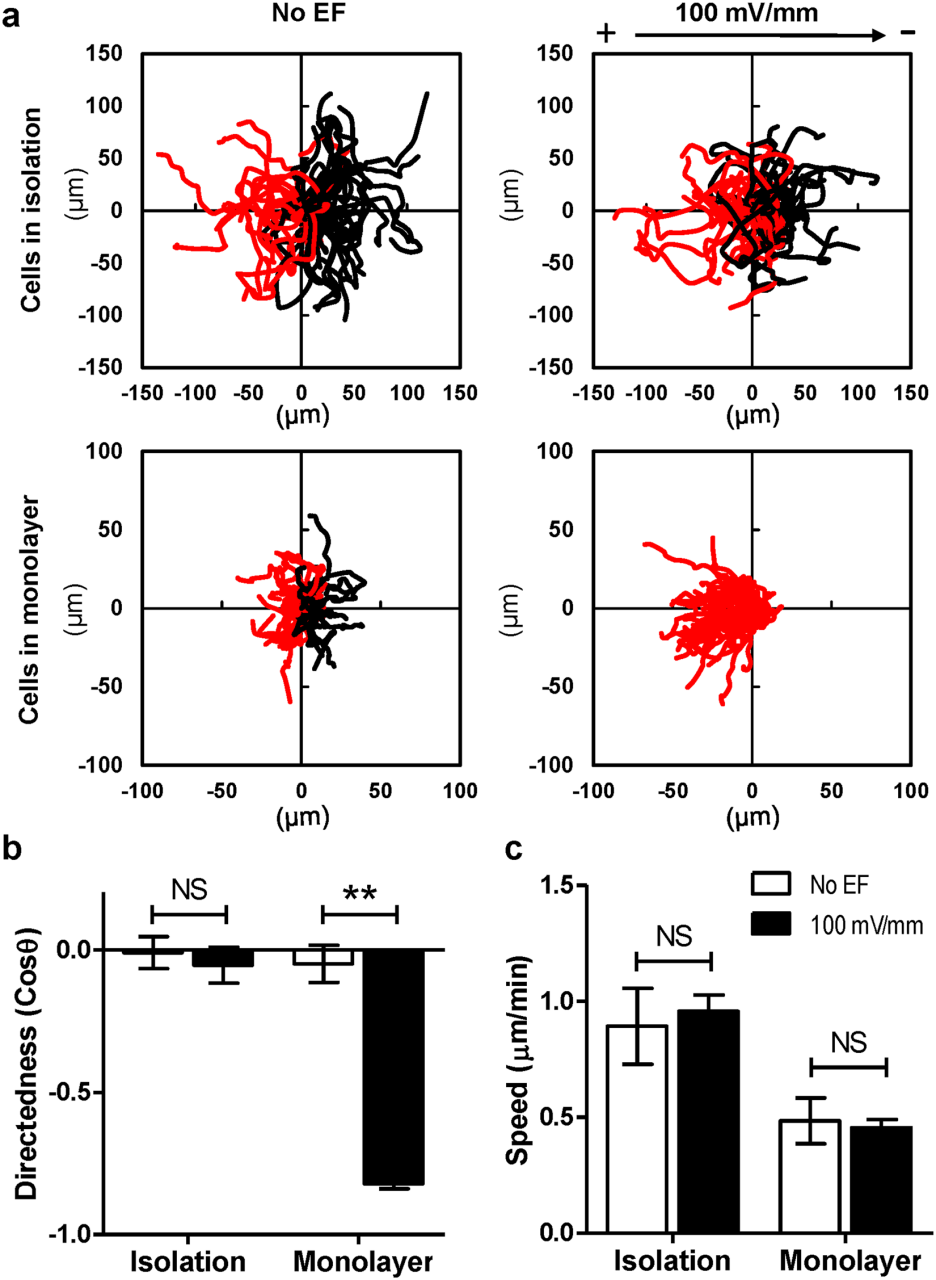
Robust electrotaxis of breast cancer cells in monolayer, not in isolation. (**a**) Cell migration trajectories of isolated cells and monolayers from one representative experiment were plotted with a common origin. Black and red lines indicate trajectories of cells migrating toward cathode and anode (or left and right in no EF controls), respectively. (**b**,**c**) Directedness and migration speed of isolated cells and monolayers in a 100 mV/mm EF. Data are shown as mean ± SEM of three independent experiments. **P < 0.01, student-t test, compared with its no EF control.

Next, we sought to clarify whether breast cancer cells respond to the electric fields that naturally exist in the tumor microenvironments. Parental 4T1 cells were seeded in galvanotaxis chambers with different density to perform migration assays *in vitro*. 100 mV/mm EF was applied to the cells and time-lapse images were recorded. As shown in Fig. 3a, parental 4T1 cells in isolation showed random migration in the absence or presence of EF, whereas, cells monolayers in confluent culture responded to the 100 mV/mm EF and migrated to the anode collectively (Supplemental Movie 1). Cells in monolayer showed a significant higher directedness value (−0.86 ± 0.13, *P* < 0.001) in the EF when compare to the no EF control (0.06 ± 0.79) (Fig. 3b). In addition, cell migration speed was not affected by EF stimulation (Fig. 3c).

### Metastatic sublines showed different galvanotaxis threshold

**Figure 4:**
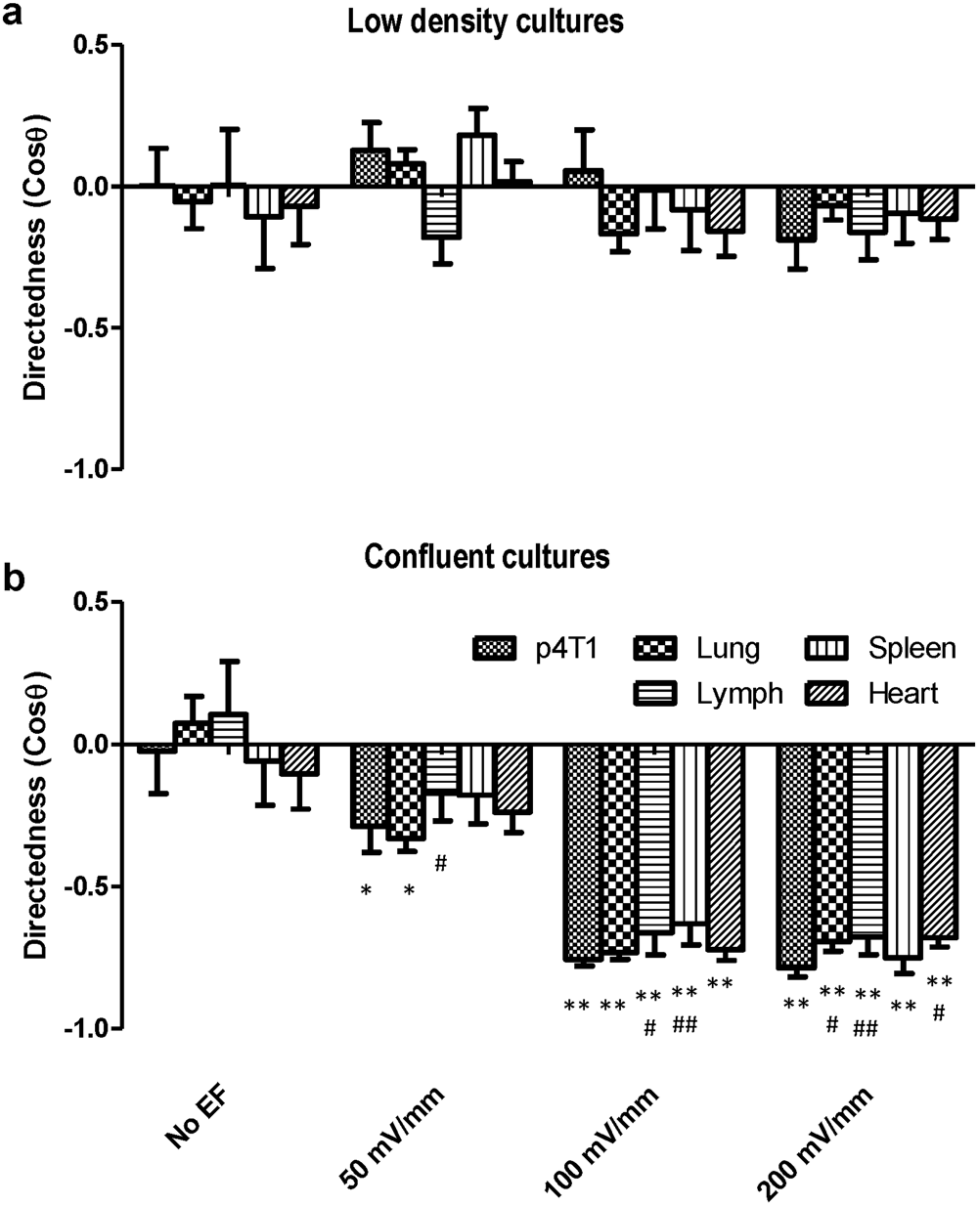
Electrotactic response of metastatic sublines to electric fields of physiological strength. (**a**) Parental 4T1 cells and cancer cells purified from metastatic sites in different organs, when cultured in very low density, didn’t show significant directional migration in EFs. (**b**) Cancer cells, in confluent culture, showed significant anodal galvanotaxis in EFs. Data are shown as mean ± SEM and compared using one-way ANOVA followed by Dunnett’s test. *p < 0.05, **p < 0.01 when compared with the no EF controls; ^#^p < 0.05, ^##^p < 0.01 compared with parental 4T1 cells of the same condition.

To evaluate the galvanotactic responses of cancer cells metastasized to different organs, we delivered 4T1-Red-FLuc-GFP cells intravenously through the tail vein then isolated 4T1 cells from metastatic tissues 4–6 weeks post-injection and established 4T1 metastatic sublines (m4T1). Cells were then seeded in galvanotaxis chambers and tested in EFs. We established 8 sublines from metastatic sites, including 4 sublines from lung, 2 from heart, 1 from axillary lymph node, and 1 from the spleen. Parental 4T1 cells were used as a control. The cells were exposed to electric fields of 50, 100, and 200 mV/mm in parallel experiments. As shown in Fig. 4a, parental 4T1 cells and metastatic sublines did not respond to electric fields of physiological strength when cultured in a low density with the directedness values close to 0. However, cells in confluent cultures responded to electric fields and migrated to the anode. Parental 4T1 and lung metastatic sublines could respond to EF as low as 50 mV/mm (*p* < 0.05 compared with its no EF control), while other metastatic sublines from lymph node, spleen, and heart showed weaker responses. The parental cells and all metastatic sublines showed significant anodal migration in a field equal to or greater than 100 mV/mm (Fig. 4b, *p* < 0.01 compared with its no EF control). When compared to the parental cells, metastatic sublines isolated from lymph node showed significant weaker galvanotaxis in monolayers (*p* < 0.05 when exposed to 50 mV/mm or 100 mV/mm EF; *p* < 0.01 in 200 mV/mm EF). In addition, the directedness of the monolayers of spleen-sublines was significantly lower when compare to the parental 4T1 cells in an EF of 100 mV/mm (Fig. 4b, *p* < 0.01), while that of the lung and heart sublines were significantly lower in an EF of 200 mV/mm (Fig. 4b, *p* < 0.05).

**Figure 5:**
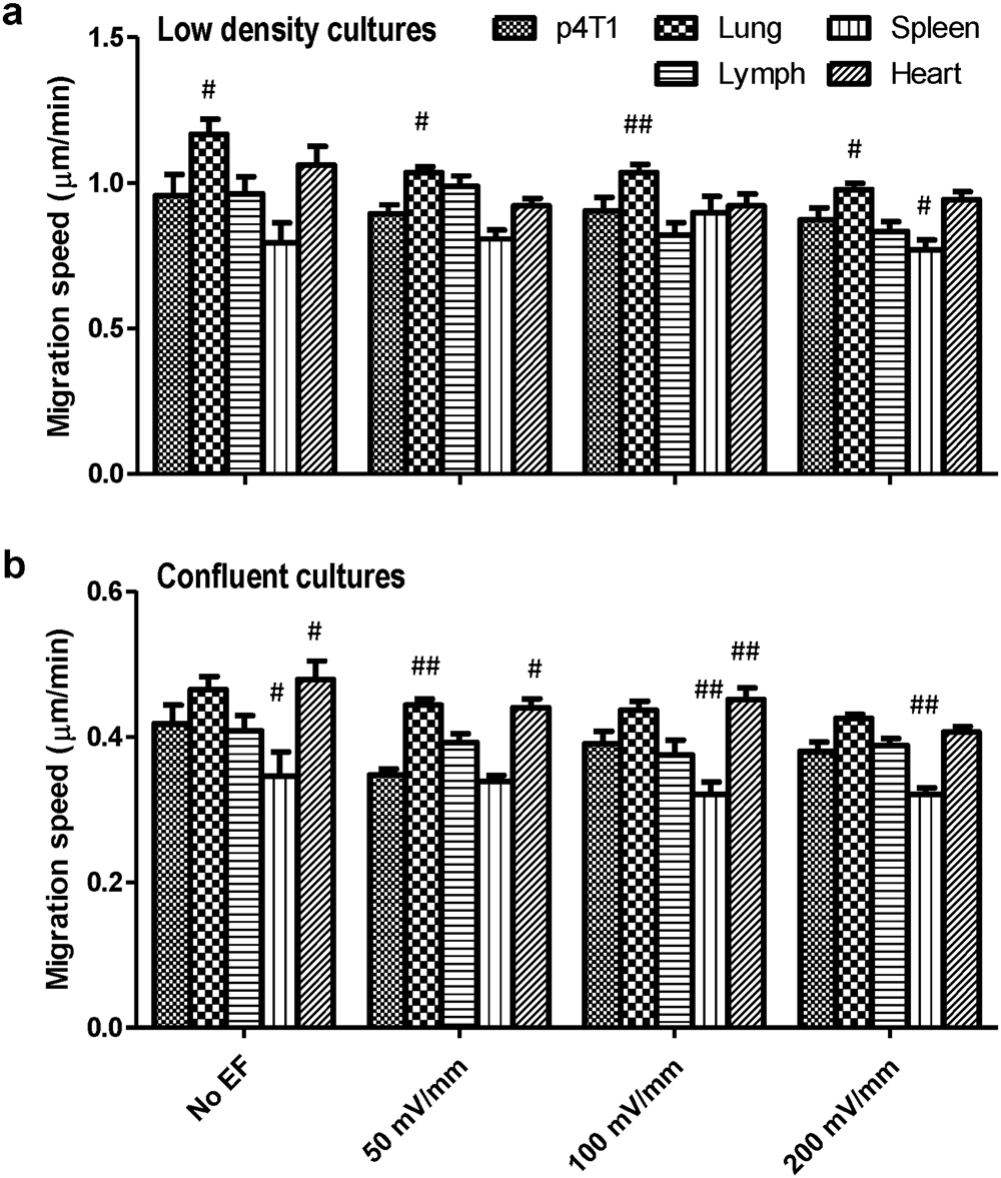
Migration speeds of 4T1 and its metastatic sublines in EFs of physiological strength. (**a**) Migration speed of parental 4T1 cells and cancer cells purified from metastatic sites in different organs. (**b**) Migration speed of cancer cells in confluent cultures. Data are shown as mean ± SEM. ^#^p < 0.05, ^##^p < 0.01 compared with parental 4T1 cells of the same condition.

In addition, the migration speeds varied among metastatic sublines, but the cells showed a similar pattern in different culture densities. The lung-sublines migrated significantly faster in isolation in the absence or presence of EFs (Fig. 5a), while the heart-sublines migrated significantly faster in monolayer (Fig. 5b). Metastatic cells isolated form spleen showed lower migration speed in most of the conditions (Fig. 5a, *p* < 0.05 in 200 mV/mm EF; Fig. 5b, *p* < 0.05 in no EF control and *p* < 0.01 in 100 mV/mm or 200 mV/mm EF). Migration persistence, namely the ratio of displacement to trajectory length, was also used to evaluate the capacity of cancer cells in maintaining the locomotion direction. As shown in sFig. 2, cancer cell monolayers have higher migration persistence in EFs (100 mV/mm and 200 mV/mm) than that of isolated cells, which suggested that cancer cells migrated more linearly in a certain direction with less turns when responding to EFs in a collective mode.

## Discussion

In the present study, we measured the endogenous EFs at breast cancer allografts *ex vivo* using a non-invasive vibrating probe and glass microelectrode. We demonstrated that the EFs naturally exist at the tumor surface, the direction and magnitude of these currents are inhomogeneous which may be due to the heterogeneity of local tumor tissue. We further tested the galvanotactic responses of the breast cancer cell line and its metastatic sublines in EFs of physiological strength and found that weak applied EFs induced significant collective migration of 4T1 cells, and the metastatic sublines showed different galvanotaxis threshold with some subtle differences.

All cells are able to generate bioelectric signals through their plasma membrane, endogenous EFs thus naturally exist in our body^14^. Much progress has been made in the EFs enhanced wound healing since it was first reported by Emil Du Bois-Reymond in frog skin wounds about 150 years ago^16^, however, there is still limited scientific understanding on the tumor EF and its role in cancer progression. Burr^32^ first reported in 1941 that tumor growth in mice disturbed the voltage gradients across the chest. He found that fast-growing tumor produced a considerable disturbance in the electric fields, whereas slow-growing tumor produced a similar disturbance over a longer period. Later on, several studies demonstrated that the surface electrical potential measurement could be used as a new diagnostic technique for breast cancer^19,20,33^. These all revealed that the bioelectric characteristics of cancer tissue differs from normal tissue and may change during cancer development. In this study, we detected electric currents of 0–10 μA/cm^2^ around a tumor, which is comparable to the measurements in corneal or skin wounds of experimental animal models^15,34^. Both outward and inward currents could be detected. We also used glass microelectrode to detect the ITP difference at tumors, and an EF of 30-465 mV/mm was detected. One could predict that these EFs exist within tumors and between cancerous and normal tissues, thus one or more electrical circuits may exist. Breast cancer is a heterogeneous disease due to the genetic heterogeneity, unstable epigenetic landscape, unstructured and disorganized microenvironment which lead to a highly variable cellular phenotype^35,36^. In our study, the electric currents and ITP measurements varied or even reversed among different positions suggested that local tumor tissues have diverse electrical activities and the tumor heterogeneity may offer a potential explanation for it. In addition, tumor size is an important factor in breast cancer staging. Studies have reported a correlation between primary tumor size and the likelihood of metastasis^37,38^. We showed that tumor EFs appear to increase with the tumor growth in size (sFig. 1) which suggested that it may serve as an index for breast cancer diagnosis.

The fact that electrical currents/fields are present at both wounds and cancers tempts us to speculate that electrical abnormalities could be an important factor shared by these two pathologies. In fact, cancers have been described as ‘wounds that never heal’ for decades^39,40^. Such electrical similarity easily match those well recognized similarities between wounds and tumor growth: the phases of pathology; macrophage polarization and activities; myofibroblasts in tumor stroma; endothelial cell and pericyte reprogramming; epithelial reprogramming; tumor microenvironment; epigenetic reprogramming and cellular plasticity; and gene expression signature^41–45^. Local electrical currents/fields shared by tumor and wounds warrants further investigation to determine its causal vs. correlative roles in different stage of tumor progression.

Endogenous EFs have emerged as an overriding signal that directs cell migration during wound healing and development^14,16,21^. These EFs are produced by directional flow of charged ions (Na^+^, Cl^−^, K^+^, Ca^2+^ and others)^46,47^ through ion channels and transporters on the cell membrane, which were found to be aberrantly expressed in many types of human cancers. They regulate different aspects of cancer cell behavior and are now considered novel cancer biomarkers^48^. Ion channels have been implicated in breast cancer proliferation and metastasis, for example, transient receptor potential channels and voltage-gated K^+^ channels could promote breast cancer cell migration and play a critical role in the development and progression of breast cancer^49,50^. The ion transport mechanisms thus have been suggested to be novel mechanisms driving the cancer process which could also offer novel clinical possibilities^51,52^. Moreover, several types of cancer cells have been tested in applied EFs of physiological strength *in vitro* and showed diverse galvanotaxis responses^23,53,54^. For instance, highly metastatic lung cancer cells showed significantly higher migration directionality and speed than low metastatic lung cancer cells^28^. Prostate cancer cell lines with different metastatic potentials, Mat-LyLu cells (strongly metastatic) and AT-2 cells (weakly metastatic), migrated to opposite directions in EFs^53^. In our study, we used the 4T1 breast cancer cells as the parental cells and injected cells through the tail vein to generate metastatic tumors. All metastatic sublines showed anodal galvanotaxis when grow in monolayer. Metastatic sublines isolated from lymph node showed significant weaker galvanotactic responses in EFs of all voltages, while lung- and heart-sublines in 200 mV/mm EF and spleen-subline in 100 mV/mm EF showed lower directedness respectively when compared with the parental cells of the same condition. Our results support the hypothesis that tumor endogenous EFs could serve as a guidance cue to direct breast cancer cell migration, and the differences of galvanotaxis threshold between metastatic subpopulations imply that abnormal sensing of weak field may have impact on local invasion to help initiate metastatic dissemination. To what extent such a mechanism contributes to metastasis and the underlying molecular pathways will be important future research.

Furthermore, collective cell migration is relevant for many processes in morphogenesis, tissue repair and regeneration, and cancer metastasis. It is prevalent in many cancers in which cells are not completely de-differentiated, including breast cancer^55^. However, the mechanisms of collective cell movement in cancer are less well studied to date compared with embryogenesis and regeneration, since cancer metastasis is a slow and long-term process. Endogenous EF has various effects including stimulation of the migration of many cell types including fibroblasts, epithelial and endothelial cells, as well as cancer cells^56^. We here showed the collective galvanotaxis of cancer cells which gives us a new understanding of the role of the tumor EFs. In addition, most epithelial cancers display the hallmarks of collective invasion into surrounding tissues, e.g. E-cadherin^55,57^. E-cadherin is a well-known tumor suppressor protein. The loss of E-cadherin expression in tumor cells, which frequently occurs during tumor progression, is believed to be one of the important mechanisms that promote cells to dissociate from the primary tumor, invade surrounding tissues, and migrate to distant sites^58^. However, metastatic cancer tissues often retain E-cadherin expression^59^, and it greatly contribute to the metastatic spread of breast cancer as E-cadherin is involved in collective cell migration during invasion and metastasis^60,61^. We previously reported that E-cadherin plays an essential role in collective galvanotaxis of large epithelial sheets^62^, blocking E-cadherin function abolished the anodal galvanotaxis of cell monolayers. Here, we demonstrated that 4T1 breast cancer cells and metastatic sublines could only respond to the EFs collectively rather that separately. This EF-promoted collective migration may relate to cancer metastasis during tumor development, and E-cadherin may play a vital role in it.

In conclusion, our results revealed that the tumor indeed generate an electric field at the CDA tumor surface, and tumor EFs increase with the size of tumors. The direction and magnitude of the electric currents at the tumor surface are non-homogeneous. Monolayer cancer cells responded to weak applied EFs of physiological strength, while cells in isolation did not. Metastatic sublines of 4T1 cells isolated from different organs also showed significant galvanotactic movement in EFs with subtle differences, which may have impact on local invasion to help initiate metastatic dissemination and colonization.

## Materials and Methods

### Animals

All the animal procedures were performed in accordance with the National Institutes of Health (NIH) Guide for the Care of Use of Laboratory Animals and approved by Institutional Animal Care and Use Committees (IACUC) of the University of California, Davis and Lawrence Livermore National Laboratory (protocol nos. 20722 and 261). Eight- to ten-week-old immunodeficient mice (NOD.Cg-PrkdcscidIl2rgtm1Wjl/SzJ (NSG), Jackson Laboratories, Bar Harbor, ME, USA) were used in this study.

### Cell line derived tumor allograft (CDA) mouse model

The transgenically modified murine mammary carcinoma cell line (Bioware Brite Cell Line 4T1-Red-FLuc-GFP) was obtained from Perkin Elmer and used in this study. 4T1 cells were cultured in RPMI 1640 (Gibco, catalog no. 11875093, Thermal Fisher Scientific Inc.) containing 10% fetal bovine serum (FBS) (Gibco, catalog no. 10437028) and antibiotics (Gibco, catalog no. 15140122). Cells then were collected and delivered to the NSG mice subcutaneously and monitored for tumor progression via palpation of the primary tumor for 3–4 weeks. Mice were housed in a temperature-controlled environment (22 ± 0.5 °C) with a 12 h-light-dark cycle and allowed free access to food and water. All efforts were made to minimize animal suffering and reduce the number of animals used. Replicates were generated from two independent mouse cohorts.

### Vibrating probe measurement

Tumor current measurement using vibrating probe was performed as previously described with minor changes^63^. The probes, platinum-electroplated at the tip, vibrated at a frequency between 100–200 Hz. Prior to measurements, probe was calibrated in mouse Ringer under experimental conditions with a current density of 1.5 μA/cm^2^. When palpable tumors are formed, mice were sacrificed and tumors were dissected and used for examining electrical properties. Under a dissecting microscope, subcutaneous tumors were secured in the non-conductive measuring chamber with the skin-side down. Reference values (baseline) were recorded with probe away from tumor (~1 cm). Plane of probe vibration was perpendicular to the tumor surface at a distance of ~5–10 µm. Current was recorded until plateau peak reached (<1 minute) in the regions of interest – the four cardinal positions of each tumor. Measurements occurred at room temperature. Data were acquired and analyzed using WinWCP V4 (Strathclyde Electrophysiology Software) and analysed using Excel.

### Glass microelectrode measurement

ITP was measured invasively by glass microelectrode impalement through the tumor surface as previously described^64,65^. Briefly, borosilicate glass capillaries without filament were purchased from World Precision Instruments (WPI, Inc., Sarasota, FL, USA; catalog no. TW150-4), and two-step heat-pulled using a Narishige PC-10 electrode puller. Microelectrode (1–2 µm tip diameter; NaCl 3 M electrolyte) that has resistances of ~1–2 MΩ was then placed onto a holder (Warner Instruments, Holliston, MA, USA) containing an Ag/AgCl wire immersed in the NaCl solution. Using the micro-positioners, the tip of the microelectrode mounted on the holder was immersed into the mouse Ringer’s solution. Tumor nodules were secured in the non-conductive measuring chamber and electrode potential offset to 0 mV prior to impalement. The tumor tissue was impaled with microelectrode for 50 µm and ITP was recorded for 1 minute in the same positions as for the current measurement. Since the tumor surface needs to be penetrated by the glass electrode tip which may change the surface potential of the original site, three repetitive measurements were done at nearby locations. Data were acquired (sampling of 100 Hz) and extracted using pClamp 10 (Molecular Devices) and treated using Excel.

### Isolation of metastatic sublines

1 × 10^6^ 4T1-Red-FLuc-GFP cells were injected into the tail vein. Due to high immunogenicity of GFP protein in immunocompetent BALB/c animals, cancer cells are cleared by the immune system and tumors do not efficiently form in this strain, NSG animals were utilized for this study to permit the use of a system that allows the exclusive isolation of metastatic cancer cells. Cancer cells were allowed to grow in those NSG-mice for 4–6 weeks to allow for metastatic tumors. Upon 6 weeks or moribund behavior of the mice, animals were euthanized via CO_2_ inhalation then necropsied for observable metastasis. Organs containing metastatic tumors were dissected then homogenized to single cell by first passing the tumor through a syringe without a needle followed by a 1 h digest with shaking at 37 °C in 100 µg/mL DNaseI (Roche, catalog no. 11284932001), 300 U/mL collagenase/ 100 U/mL hyaluronidase (STEMCELL Technologies, catalog no. 07912), 0.6 U/mL Dispase II (Roche, catalog no. 4942078001) in DMEM/F12 (Gibco, catalog no. 11320033) with 10% FBS. Digests were filtered through a 100 µm cell strainer prior to debris removal (Miltenyi Biotec, catalog no. 130-109-398) and resuspended in BD FACS Pre-Sort Buffer (BD, catalog no. 563503) prior to cell sorting on a BD FACS Melody based on GFP gating generated from cell line fluorescence and WT 4T1 cells. Isolated GFP + subpopulations were cultured in complete RPMI media and resorted for GFP expression to ensure a pure population of metastatic cells and assayed for fluorescent intensity via flow cytometry prior to galvanotaxis experiments (sFig. 3).

### Galvanotaxis assay

Galvanotaxis experiments were performed as previously described^66^. Briefly, silicone stencils with 9 wells were placed in the galvanotaxis chambers for multi-spot seeding, which allowed us to simultaneously examine the galvanotactic responses of multiple sublines in the same chamber. Cancer cells isolated from different organs were then seeded singly to the wells with ideal density. 200 µl cell suspension were added in each well at concentrations of 2.2 × 10^4^/mL and 4.5 × 10^5^/mL for low density culture (~150 cells per mm^2^) and confluent culture (~3 × 10^3^ cells per mm^2^), respectively. Cells were cultured overnight at 37 °C with 5% CO_2_ to allow sufficient attachment. Before EF stimulation, the stencils were lifted. Unattached cells were removed and fresh RPMI 1640 medium with 10% FBS and antibiotics were added. Current was applied to the chamber through agar-salt bridges connecting with silver/silver chloride electrodes in Steinberg’s solution as described previously^67^. Agar gel was pre-prepared in a sterilized condition by dissolving 3% (wt/vol) agar powder (Sigma, catalog no. A1296) into Steinberg’s solution^67^. 5 mL medium were added into reservoirs to ensure salt bridge contact and support cell viability during EF stimulation. A pair of reference electrodes connecting to a digital multimeter was placed in the two reservoirs to monitor EF strengths at the beginning of the experiment and every 30 minutes afterward to ensure consistent EF application. Cell migration was observed with a Carl Zeiss Observer Z1 inverted microscope with MetaMorph NX program (Molecular Devices, Sunnyvale, CA, USA). The microscope system was able to record serial time-lapse images of multiple locations on the multi-channel galvanotaxis chamber simultaneously. A 10× phase contrast objective lens was used for microscopy. Images were taken at 5-minute intervals for 3 hours.

### Quantification of cell migration

Cell migration was analyzed to determine directedness (cos θ) and trajectory speed by using ImageJ software from the National Institutes of Health (http://rsbweb.nih.gov/ij/) with MTrackJ and Chemotaxis tool plugins as previously described^14,68^. The position of a cell was defined by its centroids. Cells that divided, moved in and out of the field, or merged with other cells during the experiment were excluded from analysis. Directedness was used as an indicator of galvanotaxis which is defined as cosine of the angle between the EF vector and a straight line connecting the start and end positions of a cell. A cell migrating directly toward the cathode would have a directedness of 1 while a cell migrating directly to the anode would have a directedness of −1. Migration speed is the trajectory distance divided by time, and migration persistence is the ratio of displacement distance (the straight-line distance between the start and end positions) to trajectory length traveled by a cell. The persistence would be equal to 1 when cells move persistently along a straight line in a given direction. For each condition, at least 50 cells were analyzed. All experiments were repeated, and data in the bar charts were averaged from three replicates.

### Statistical analysis

All data are represented as means ± standard error of the mean (SEM). Statistical analyses were performed using GraphPad Prism 7.0 with one-way ANOVA followed by Dunnett’s test or unpaired two-tailed Student’s t-test. For correlation analysis between tumor weight and electric current density, the Pearson’s correlation coefficient was computed. *P* value was set at 0.05 for rejecting null hypotheses.

## Supplementary information

Supplementary information.

Supplementary information 2.
