# Supplementary material for: Electric Fields at Breast Cancer and Cancer Cell Collective Galvanotaxis
Source: Sci Rep. 2020 May 26;10:8712. doi: 10.1038/s41598-020-65566-0 (PMC7250931; doi:10.1038/s41598-020-65566-0)
Supplement: Supplementary file 1 — Supplementary information. [file 41598_2020_65566_MOESM1_ESM.pdf]

Supplemental Figures

Electric Fields at Breast Cancer and Cancer Cell Collective Galvanotaxis

Kan Zhu[1], Nicholas R. Hum[2,3], Brian Reid[1], Qin Sun[1,4], Gabriela G. Loots[2,3]*, and Min Zhao[1]*

[1] Institute for Regenerative Cures, Departments of Dermatology, Department of Ophthalmology & Vision Science, School of Medicine, University of California, Davis, CA 95616

[2] Physical and Life Sciences Directorate, Lawrence Livermore National Laboratory, Livermore, CA 94550

[3]University of California, Merced, School of Natural Sciences, Merced, CA.

[4] School of Life Science, Yunnan Normal University, Yunnan, China

*** Correspondence:** MZ (minzhao@ucdavis.edu) or GGL (loots1@llnl.gov)

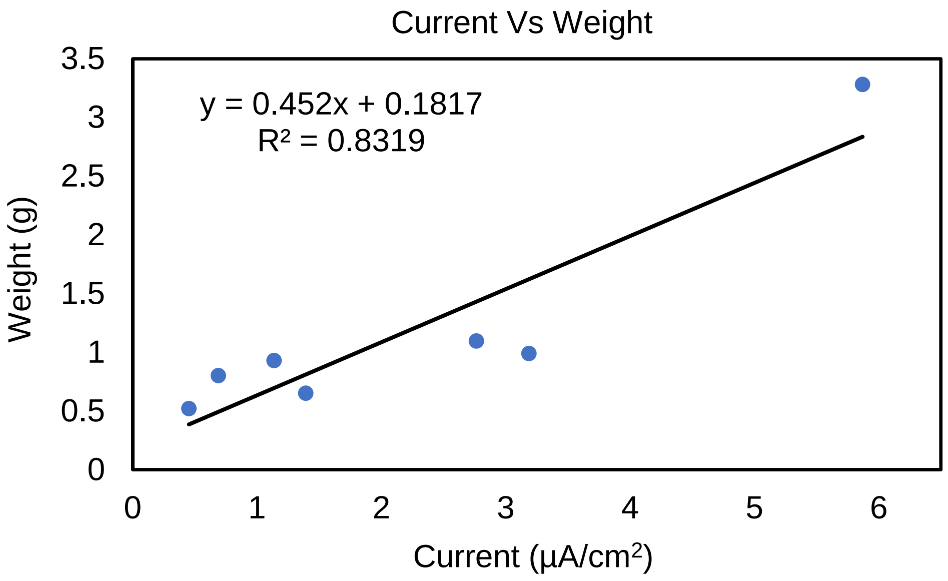

sFig. 1. Correlation between Tumor weight (size) and electric current density. Compilation of all tumors tested. Data of current magnitude are from Fig. 1d. Solid line: linear regression (r^2=0.8319); r: correlation coefficient (P=0.0042).

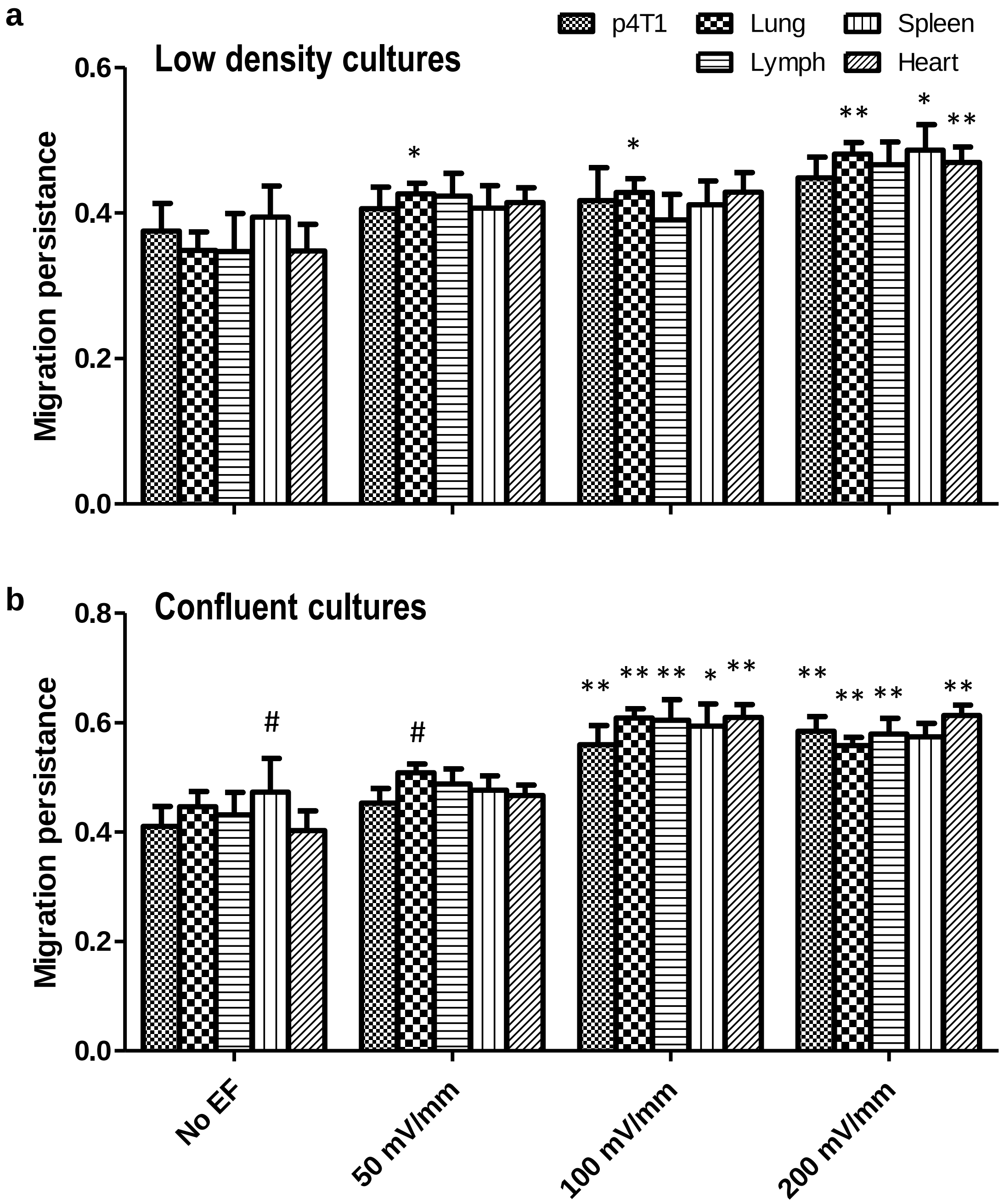

sFig. 2. Migration persistence of metastatic sublines in EFs. (a) Cells in isolation; (b) Cells in monolayer. At least 50 cells were analyzed for each condition. Data are shown as mean ± s.e.m. * $p < 0.05$, ** $p < 0.01$ compared with its no EF control; # $p < 0.05$, ## $p < 0.01$ compared with parental 4T1 cells of the same condition.

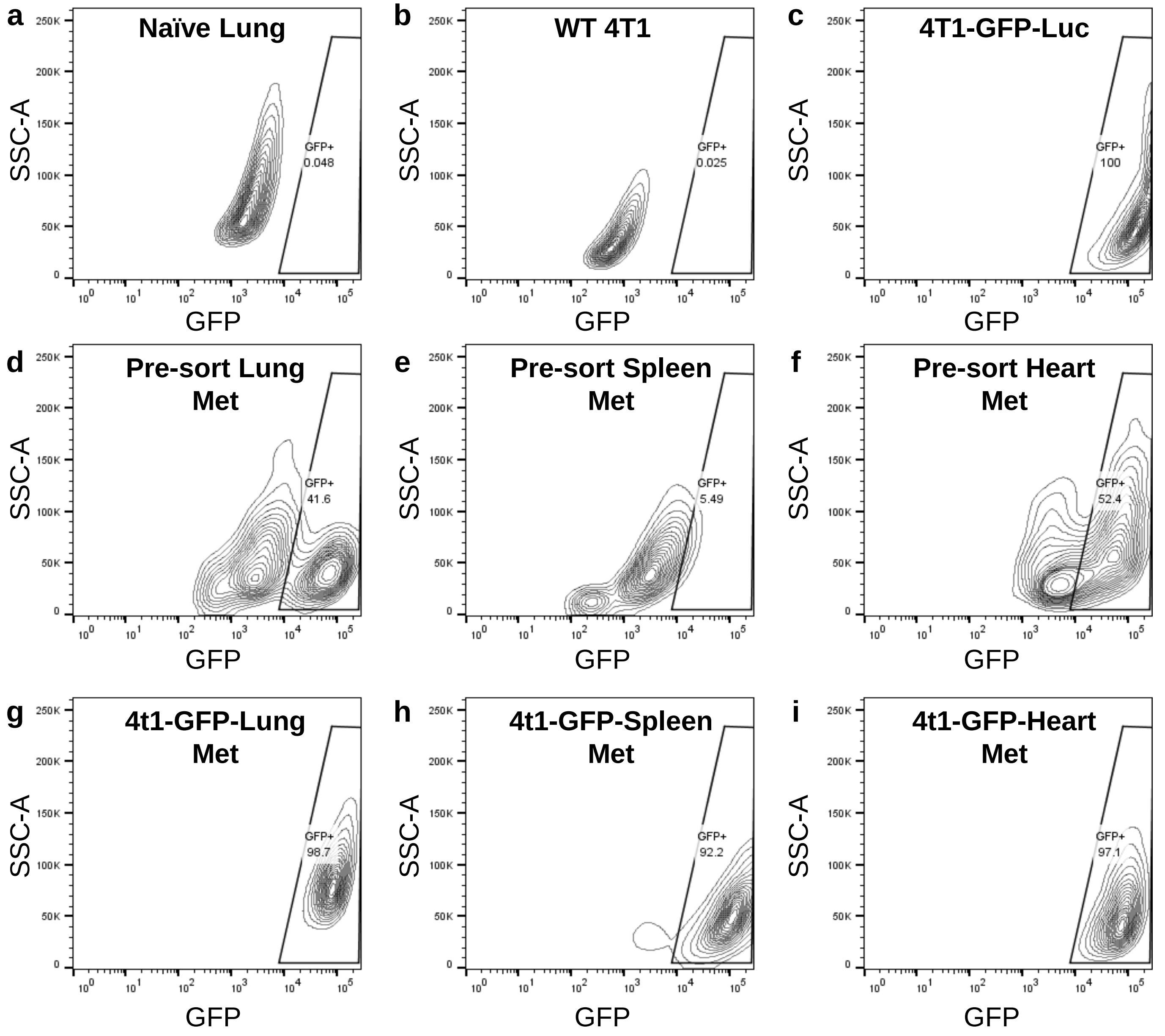

sFig 3. 4T1-GFP metastatic subline generation. Cytometric analysis of GFP intensity of (a) naïve lung tissue, (b) WT 4T1, (c) 4T1-GFP-Luc in culture, (d-f) primary cell populations from tissues of interest, (g-i) established metastatic cell lines following 2 FACS isolations
